# Endophytic Bacterial Microbiome Diversity in Early Developmental Stage Plant Tissues of Wheat Varieties

**DOI:** 10.3390/plants9020266

**Published:** 2020-02-18

**Authors:** Jana Žiarovská, Juraj Medo, Matúš Kyseľ, Lucia Zamiešková, Miroslava Kačániová

**Affiliations:** 1 Department of Genetics and Plant Breeding, Faculty of Agrobiology and Food Resources, Slovak University of Agriculture in Nitra, Tr. A. Hlinku 2, 94976 Nitra, Slovakia; xkysel@uniag.sk (M.K.); xzamieskova@uniag.sk (L.Z.); 2 Department of Microbiology, Faculty of Biotechnology and Food Sciences, Slovak University of Agriculture in Nitra, Tr. A. Hlinku 2, 94976 Nitra, Slovakia; juraj.medo@uniag.sk; 3 Department of Fruit Sciences, Viticulture and Enology, Faculty of Horticulture and Landscape Engineering, Slovak University of Agriculture, Tr. A. Hlinku 2, 94976 Nitra, Slovakia; miroslava.kacaniova@uniag.sk

**Keywords:** wheat, drought, endomicrobiome diversity

## Abstract

Endophytic bacteria are an important part of different functions in plants that lead to plants’ production characteristics as well as their stress response mechanisms. Endophytic bacterial diversity was analyzed in this study to describe 16S rRNA variability and changes in the leaves of drought-tolerant and drought-susceptible wheat when growth under in vitro conditions. A metagenomic analysis was applied and a pilot exploratory study was performed to prove this type of analysis as applicable to tracking endophytic bacterial diversity changes when a drought stress is applied to an in vitro culture of wheat. The study showed that the changes in the bacterial endophytes’ variabilities associated preferentially with the drought stress varietal characteristics of the analyzed wheat instead of the applied stress conditions.

## 1. Introduction

Plant-associated microorganisms are referred as the plant microbiome. Bacterial communities together with other microbial inhabitants are an inevitable and abundant part of all of plants, however in leaves they face temperature fluctuations and ultraviolet radiation and their occurrence is limited by their access to nutrition [1]. Endosymbiotic bacteria have been reported as one of the factors that enhance the absorption of nutrients with a direct effect on the growth of plants [2]. The plant microbial community has a broad-spectrum of species representation. The largest portion is represented by microscopic fungi [3], followed by bacteria [4]. Species of Archea, yeast, Protista or other non-classified unicellular organisms may occasionally appear in such communities, too [5]. According to [6], each bacterial organism fluctuates between the internal space and plant surface during its colonization. The mechanisms of persistence and the survival of microorganisms within plant tissues are still not fully understood. It should be noted that most competitive endophytes can migrate between different types of plant tissues or different locations on the same type of tissue in the internal environment of the plant [7]. The specific diversity and species spectrum of endophytes are related to various factors that can be classified into four groups: geography and climate, soil, multitrophic interactions and natural and anthropogenic disorders. 

It is relatively unusual for a plant to be colonized by only one type of endophyte. The high number of species and high amount of microbial biomass in small populations suggest frequent multitrophic interactions between bacteria, micro- and macroscopic fungi, microfauna, plants and the environment [8,9,10].

Many well-defined microbiologic methods exist to analyze bacterial spectra in the soil, but methods of analyzing culture-dependent endophytic bacteria are still limited [11]. Gene sequences of 16S rRNA provide a great source of information on the occurrence of bacteria both in soil as well as in plant tissues [12]. The 16S rRNA genes contain a conserved region that can be matched by universal/specific designed metagenomic primers as well as hypervariable regions that permit a very specific differentiation of bacteria in the sample [13]. Using NGS platforms has provided a powerful alternative for the analysis of microbial communities when compared to the sequencing of 16S rDNA for cultivated bacterial isolates or libraries of clones [14]. 

Analysis of the wheat endophytic microbiome has been successfully performed by different authors in regards to rhizosphere endophytes, but metagenomic-based studies are still rare. Analysis of wheat rhizosphere resident genetic diversity was performed in [15]. In that study, 30 operational taxonomic units including the classes Alfaproteobacteria, Betaproteobacteria, Deltaproteobacteria, Gammaproteobateria, Actinobacteria, Bacilli, Clostridia and uncultivable bacteria were reported. The wheat rhizosphere was found here to have common rhizospheric or bulk soil bacteria, of which Pseudomonas, Stenoprophomonas and Bacillus were the most abundant. Another rhizosphere endophytes study was aimed at two cultivars of wheat, i-e Freed-06 and Chakwal-50, during the flowering stage [16]. The rhizosphere microbiome exhibited microbial communities representing 8 phyla, 14 classes, 14 orders, 23 families and 33 genera of endophytes.

In this study, a metagenomic high-throughput sequencing approach of 16S rRNA variability analysis was used for the characterization of endophytic bacteria diversity and its changes in leaves of drought tolerant and drought susceptible wheat. A metagenomic approach was applied and a pilot exploratory study was performed to prove this type of analysis as applicable to tracking endophytic bacterial diversity changes when drought stress is applied to an in vitro culture of wheat.

## 2. Material and Methods

### 2.1. Plant Sampling and in Vitro Drought Stress

Application of drought stress conditions was performed in vitro by PEG 6000 under different drought stress intensities (0 and 5 and 10%; w/v) in MS growth medium [17]. Polyethylene glycol (PEG) was used as an osmotic substance to induce water stress on plant tissues. Wheat seed sterilization was realized in 70% ethanol (v/v) during 50 s when shaken, then 3% NaHClO (v/v) was applied for 20 min and final rinsing in dH2O was performed. Murashige and Skoog (1962) medium was used in a volume of 70 mL and a total of five wheat seeds were placed into one bottle. Bottle duplicates were used for each wheat variety and all plants were grown in a single experiment. Seed germinating was synchronized at the temperature of 4 °C and dark conditions in the growth chamber. Seven-day old sprouts were cultivated under the conditions of 23 °C, 16/8, 4,000 lux. Young leaves were collected for DNA extraction at the sixth week of growth. Young leaves of in vitro wheat plants were collected and weighted and further surface sterilization was performed immediately. All the leaves were washed briefly with hypochlorite sodium (50%) and immersed in sterile distilled water separately for 2 min. Treated leaves were rinsed three times in sterile distilled water and dried in sterile towels. They were kept at −20 °C until conducting DNA extraction from 100 mg of plant material. One factorial ANOVA was calculated for the fresh weight of collected leaves with a post-hoc Tukey HSD test. A total of four different wheat varieties were analyzed for their endophytes: Seladon (drought tolerant), Venturero (drought tolerant), Aladin (drought susceptible) and Dagmar (drought susceptible) [18].

### 2.2. DNA Extraction and Illumina Library Preparation

DNA was extracted from pooled leaf samples according to the Rogers and Bendich [19] protocol with no modification. The extracted genomic DNA was inspected for the parameters of its quantity and quality (A26/A280) by a Nanodrop NanoPhotometer (Implen). Universal bacterial primers 515F and 806R [20,21] enhanced by a 6-bp identification sequence (tag) were used for amplification of the V4 part of the 16S rRNA gene. Composition of the 30-µL PCR mixture was as follows: 1 µL (20 ng) of extracted DNA, 4 µL each primer with concentration of 0.3 µM.ml^−1^, KAPA HiFi HotStart ReadyMix (1X) (Kapa Biosystems,Wilmington, USA). DNA aliquots were PCR-amplified in a SureCycler 8800 thermal cycler (Agilent,Santa Clara, USA) with 90 s denaturation at 98 °C, 35 cycles of 15 s denaturation at 98 °C, 15 s annealing at 62 °C and 15 s elongation at 72 °C, after which a final elongation step of 120 s at 72 °C was performed. 

PCR products were visualized on agarose gels (2% in TBE buffer) containing ethidium bromide and purified with a PCR purification kit (Jena Bioscience, Jena, Germany). The concentrations of the PCR products were measured with a Qubit 2.0 Fluorometer using the HS dsDNA quantitation assay (ThermoScientific, Walthem, USA). DNA was adjusted to an equal concentration and pooled together. Illumina adapters were attached by the Truseq LT PCR-free kit (Illumina) with a modification to skipp the DNA fragmentation and size-selection steps. The library was quantified by qPCR using a NebNext Quantification kit (New England Biolabs, Ispwich, USA), diluted to 4-nM concentrations and denatured. A MiSeq Reagent Kit v3 (600-cycle) was used for sequencing. Six-hundred microliters of 20 pM library with a 1% PhiX spike was loaded into the cartridge.

### 2.3. Data Processing

Basic processing of raw sequencing data was performed by Seed 2 software [22]. Sequences were joined using the Fastq-join. Sequences with an overall quality lower than Q30 were removed from further analysis. The sequences were trimmed and assigned to individual samples based on their barcodes. Primer sequences were removed from the reads. The presence of chimeric sequences was analyzed using the Vsearch tool [23], and these were also removed from further analysis. Sequences were clustered to OUT with a level of 97% similarity using the same tool. Operational taxonomic units were used to categorize bacteria based on sequence similarity to distinguished them. OTUs with less than five members were removed from analysis as they commonly represent sequencing artefacts. The most abundant sequence in each OTU was found and identified with the aid of a Ribosomal Database Project (RDP) Classifier against the 16S rRNA database (RDP Release 11) at a confidence threshold of 70% [24]. Chloroplasts and mitochondria OTUs as well as non-identified OTUs were removed from further analysis. The OTU table and identification data were processed using the pivot table in MS Excel, then statistically evaluated in R [25]. 

Rarefaction curves were constructed in SEED2 for each individual sample, showing the number of observed OTUs relative to the number of total identified bacterial rRNA sequences. To calculate diversity indices, sequences in each sample were rarefied. The alpha diversity of OTU richness that correspond to the number of OTUs observed per sample was used together with the corrected evenness to describe how balanced each community was. Shannon and inverse Simpson diversity indices, which take in account both mentioned properties, were also computed for each sample. Indices were calculated in ComEcoPac [26]. Variation in richness and diversity among samples was assessed using ANOVA followed by a Tukey HSD. 

Venn diagrams were created using the Venn Diagram package to visualize the number of OTUs shared between cultivar types. To estimate and evaluate the changes between microbial communities in samples (beta diversity), the normalized counts were used to calculate a Bray-Curtis dissimilarity matrix, which was used to examine the similarities of the memberships and structures found in the various samples and visualize them using non-metric multidimensional scaling (NMDS) via the Vegan package in R environment [27]. 

To test if the communities were significantly different between treatments and varieties, Adonis, a nonparametric statistical method with 999 permutations, was used in Vegan.

## 3. Results and Discussion

**Figure 1:**
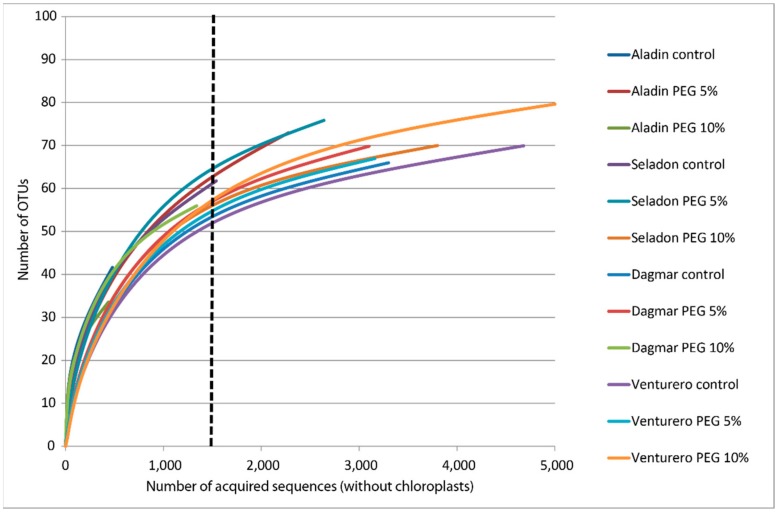
Rarefaction curves of microbial clusters in the microbiomes of different wheat varieties cultured in vitro. Rarefaction curves were assembled showing the number of operational taxonomic units defined at a 97% sequence similarity, relative to the number of total sequences. The dashed vertical line indicates the number of sequences subsampled from each sample to calculate the alpha diversity estimates.

Sequencing of the metagenomic amplicon libraries resulted in a total of 166,314 raw reads prior to quality checking. After further processing of the quality filters for the sequences with Q 30 and lengths of 280–300 bp, a total of 165,319 high-quality reads were recovered from the analyzed wheat samples. Their clustering by Vsearch and chimera elimination resulted in a total of 3338 clusters. The elimination of low members’ clusters resulted in 707 clusters. Removing of chloroplast, mitochondria and unidentified (predicted sequential artifacts) clusters resulted in a total of 113 clusters containing 32,029 true bacterial sequences. There was a range of 455 to 5114 sequences per sample. Rarefaction curves evaluating the OTU richness per sample were saturated (Figure 1), indicating that saturation of bacterial communities at the 97% OTU level was reached for the majority of samples. In some samples, low-sequence counts caused by the chloroplast sequence-removing procedure resulted in lower saturations. Sequences were normalized for the lowest amount using the simple rarify method.

Basic indices of alpha diversity were calculated from the normalized sequences. The values of the indices were compared using multifactorial ANOVA according to the interactions between varieties and drought-stress treatments. The effects on Evenness in the Shannon and Chao index were insignificant. In the case of the Simpson index, a significant effect existed for the Venturero, where this variety had a significantly higher index (Table 1, Table 2 and Table 3).

**Figure 2:**
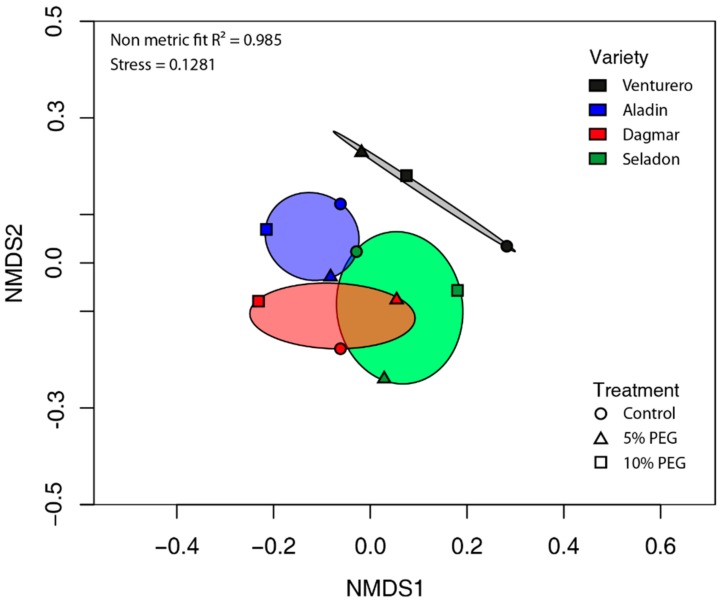
Non-metric multidimensional scaling (NMDS) plot for all samples at the OTU phylogenetic level based on the Bray–Curtis similarities of relative bacterial abundances of different wheat varieties cultured in vitro.

Beta diversity of the endophytic microbial community was described by non-metric multidimensional scaling (NMDS) analysis using Bray–Curtis distances. Here, the differences of the microbial communities of the analyzed samples were found (Figure 2). This was not confirmed further by permutation multivariate analysis of variance where the effect of variety or drought treatment was insignificant. 

**Figure 3:**
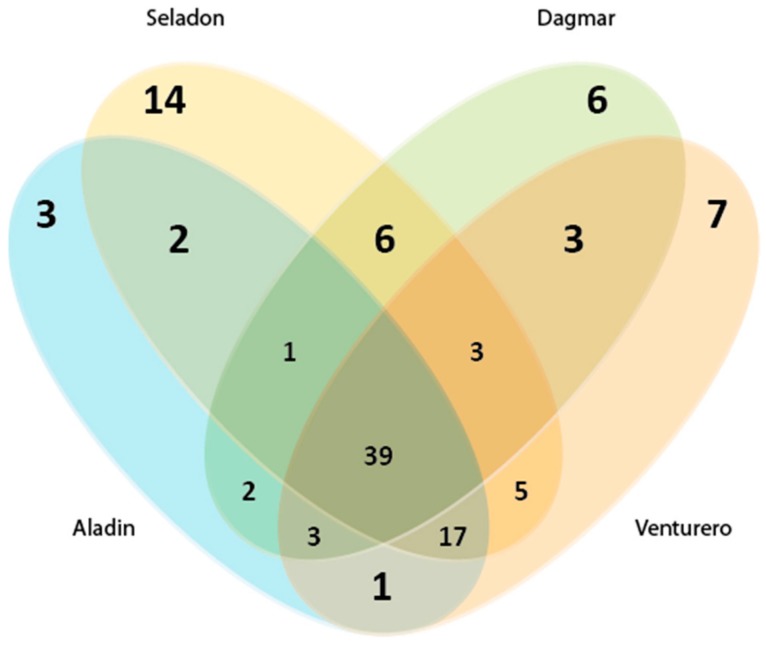
OTU distribution across the analyzed wheat varieties. Venn diagram showing the number of operational taxonomic units (OTUs) shared and unique among different wheat varieties.

Here, a traditional approach was used to cluster sequences into operational taxonomic units that reflect the phylogenetic boundaries of distinct bacterial species The most OTUs (73) were obtained for the Seladon variety, which had the highest number of unique OTUs (Figure 3). Wheat varieties Dagmar, Venturero and Aladin has 67, 64 and 58 OTUs, respectively. 

Host-associated and environmental samples always represent a mixture of different microorganisms including dead, live, vegetative, sporulated, inactive or active cells, as well as different macromolecules and cell debris. Many other substances from these samples depend on the microbial lysis protocols to be eliminated during the preparation of community DNA for microbiome testing [28]. 

Bacterial endophytes help host plants resist or tolerate different biotic or abiotic stresses by releasing specific substances, competing for space and nutrients or modulating the plant response [29]. Their niches are preferentially localized in the intracellular spaces of plants because of the abundance of nutrients. Intracellular spaces on the other side represent a barrier that dampens and modulates the direct impact of adverse abiotic stresses on the microbial communities that inhabit it. This is in agreement with our findings that the endophytic communities of different wheat varieties are relatively stable towards applied drought stress, but with differences among the varieties. An insignificant difference was calculated for the treatment–variety relationship, and well-known plant entophytic bacteria were found to be members of this community. This could be explained as a consequence of the multilevel surface sterilization of wheat seeds—together with the in vitro conditions of growth during the six weeks—that do not threaten the stability of intracellular communities of typical plant endophytes. In our study, *Burkholderia* was found to be the most abundant case in all the analyzed wheat varieties. This taxon is widely reported to be a stable part of the plant endophytic microbial community [30]. 

**Figure 4:**
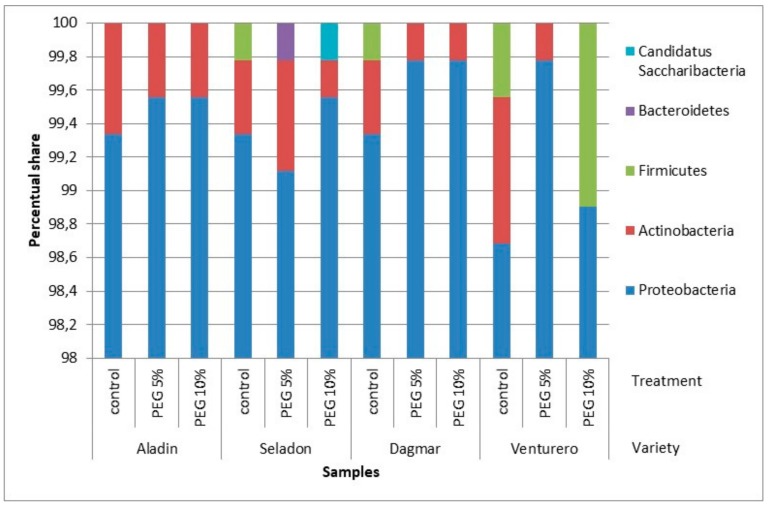
Distribution of the OTUs. Relative sequence abundance of bacterial phylla associated with the in vitro wheat varieties from pooled samples. Relative sequence abundance was calculated as the proportion of sequences belonging to a particular class of all 16S rRNA sequences recovered.

When analyzing the individual groups of bacteria, only five phyla were more abundant with the great dominance of Proteobacteria (Figure 4), and different genera were identified for individual wheat varieties (Table 4). The biggest nucleotide similarity (98–100%) was identified within OTUs for *Paraburkholderia graminis*.

Polyethylene glycol was used as an osmoticum, as the PEG molecules were too large to be absorbed by plant roots. Different PEG concentrations in the surrounding medium cause outward movement of water from the plant cells [31]. Thus, plant cells undergo an environment of water stress [32]. The osmotic stress induced by PEG indicated differences in the fresh weights of leaves from six-week-old plants under in vitro conditions as well as for individual wheat varieties, whereas treatments and endophytic microbiome–treatment interactions did not vary as much. The average fresh weight of leaves was 1.167 g for Venturero, 1. 095 for Seladon, 0.92 for Dagmar and 0.919 for Aladin. Both susceptible drought varieties were significantly different to the tolerant ones at the level of *p* < 0.01.

Endophytes and epiphytes have been seen to have a basic and key role in the effect of drought stress to the host plants. Endophytic fungi *Neotyphodium coenophialum* supports the development of roots that help the plant to use soil humidity very effectively and to better absorb nutrients. Seeds and root colonization of Bolivian Andes plants, quinoa, rice or halophyte *Limonium sinense* by bacterial endophytes of *Bacillus* sp., *Pseudomonas* sp., *Klebsiella* sp., *Serratia* sp., *Arthrobacter* sp., *Streptomyces* sp., *Isoptericola* sp. and *Microbacterium* sp. helps to accumulate substances similar to glycine and betaine that lead to a better resistance to salinity [33,34,35,36]. 

*Herbaspirilum* was the third most abundant genus found in this study. *Herbaspirilum* sp. have been reported previously [37] as a typical colonizer of wild rice. Wheat varieties were analyzed for their endophytic changes via the application of PEG 6000, which induces drought stress [38]. This was hypothesized to result in bacterial settlement changes, as the presence of bacteria supports acclimatization at low temperatures and the higher antioxidation activity of plans as a result of drought. This has been reported for *Bacillus subtilis* [39] and *Burkholderia phytofirmans* [38], but was not confirmed by our study despite the fact that both of this taxa were found in the profiles of the tested wheat varieties. *Burkholderia* sp. supports antioxidant activity and changes in the photosynthetic activity that accompanies changes in the metabolism of saccharides and substances. Its presence is active in response to drought and low temperatures through starch, prolin, phenolic compounds, glucose, saccharose, raffinose and galactinol [40,41]. Wheat variety Venturero is naturally adapted to conditions of permanently low residual moisture, which supports the finding of the bacterial settlement of *Burkholderia* sp. being the most abundant in the metagenomic results. The position of *Burkholderiales* (*Burkholderia* sp.) can even be seen in the profiles of narrow groups of genera (Table 3) where its abundance is present in all concentration levels of PEG. A detailed analysis of the individual concentration levels showed a higher representation of three specific genera (*Propionibacterium* sp., *Herbaspirillum* sp. and *Bradyrhizobium* sp.) at different PEG concentrations, respectively. The most numerous species in *Propionibacterium* sp. are mesophilic; however, they are resistant to higher temperatures and have adapted to survive. The optimum growth temperature is 30 °C, but the temperature tolerance is as high as 70 °C (for 20 s), or for some strains 76 °C (for 10 s). In general, an inhibitory effect on the *Propionibacterium* sp. is produced by high acidity, high salt concentrations, extreme temperatures and, last but not least, insufficient water activity [42]. The environment with the highest addition of PEG 6000 (10%) cannot be characterized as an extreme one, as the concentration can be further increased and the spectrum of bacterial populations can expand, but even under such conditions the bacteria species are in a condition where their activity is limited by residual humidity. Commonly numerous endophytic bacteria of *Bacillales* or *Pseudomonadales*, which also belong to the anthropogenic group [8,43], have appeared in identified settlements sporadically. Previous metagenomic studies of amaranth have shown numerous populations of these bacteria [20], while the discovery of populations on wheat have been minimal. The presence of *Bacillus* was reported previously to be related to the promotion of expression and activity of pyrophosphatase to ensure hydrogen pump cell vacuoles in peppers [44]. *Enhydrobacter* sp., *Phenylobacterium* sp. and *Corynebacterium* sp. were found sporadically in the endophytic community of the in vitro cultured wheat varieties, but all of them were reported to be a part of endomicrobiome [45,46,47,48].

## 4. Conclusions

This study showed that bacterial endophytes’ variability was associated with drought stress in four wheat varieties. Changes in the endophytes’ variabilities were recorded using a metagenomic NGS approach, and an interactive drought-stress simulation was carried out. In summary, the results indicate that there was a the susceptibility of the wheat varieties to drought but not to the PEG concentration in the growth medium, in spite of the different fresh weights of leaves when the samples were collected for the analysis. Future studies should further analyze the changes of the entophytic microbiome under drought stress and possible functional changes based on this.

## Figures and Tables

**Table 1:** Summary of alpha diversity indices for diversity among the analyzed wheat endophytes.

Variety	Treatment	Richness	Evenness	Shannon Index	Simpson Index	Chao1 Index
Aladin	control	40	0.72919	4.117665	0.076353	80.5
	PEG 5%	44	0.736746	4.276029	0.070595	62.3
	PEG 10%	35	0.758802	4.076261	0.076643	56.1
Seladon	control	44	0.712485	4.166971	0.076208	84.0
	PEG 5%	45	0.726226	4.258377	0.071281	85.0
	PEG 10%	45	0.727704	4.265035	0.072189	75.0
Dagmar	control	43	0.737963	4.251187	0.068683	88.1
	PEG 5%	46	0.730833	4.308297	0.069687	90.1
	PEG 10%	38	0.736881	4.085601	0.075793	63.6
Venturero	control	40	0.726042	4.103665	0.078382	58.7
	PEG 5%	35	0.743998	4.011629	0.077358	51.9
	PEG 10%	42	0.708634	4.08912	0.082014	69.0

PEG—polyethylene glycol.

**Table 2:** Analysis of variance for the Simpson index-Type III Sums of Squares.

Source	Sum of Squares	Df	Mean Square	F-Ratio	P-Value
MAIN EFFECTS					
A:Variety	0.00010154	3	0.0000338466	5.23	0.0411
B:Treatment	0.0000398069	2	0.0000199035	3.08	0.1203
RESIDUAL	0.0000388023	6	0.00000646705		
TOTAL (CORRECTED)	0.000180149	11			

All F-ratios are based on the residual mean square error.

**Table 3:** The 95.0% LSD analysis for the Simpson index.

Variety	Count	LS Mean	LS Sigma	Homogeneous Groups
Dagmar	3	0.0713875	0.00146823	A
Seladon	3	0.0732263	0.00146823	A
Aladin	3	0.0745305	0.00146823	AB
Venturero	3	0.0792513	0.00146823	B

Homogenous groups represented by the same letter are not statistically different; α = 0.05.

**Table 4:** Distribution of identified bacterial genera in the individually analyzed wheat varieties.

Genus	Aladin			Seladon			Dagmar			Venturero		
	0% PEG	5% PEG	10% PEG	0% PEG	5% PEG	10% PEG	0% PEG	5% PEG	10% PEG	0% PEG	5% PEG	10% PEG
Burkholderia	x	x	x	x	x	x	x	x	x	x	x	x
Propionibacterium	x	x	x	x	x	x	x	-	-	x	x	-
Herbaspirillum	x	x	x	x	x	x	x	x	x	-	-	x
Bradyrhizobium	x	-	-	-	x	-	x	x	-	-	-	x
Bacillus	-	-	-	-	-	-	-	-	-	-	-	x
Staphylococcus	-	-	-	x	-	-	-	-	-	x	-	-
Enhydrobacter	x	-	x	x	-	-	-	-	-	-	-	-
Sphingomonas	-	-	x	-	x	-	-	-	-	x	-	-
Phenylobacterium	-	-	-	x	x	x	x	-	x	-	-	-
Corynebacterium	x	-	-	-	x	-	-	x	x	-	-	-
Pelomonas	-	-	-	-	-	-	-	-	-	x	-	-
Paraprevotella	-	-	-	-	x	-	-	-	-	-	-	-
Streptococcus	-	-	-	-	-	-	-	-	-	-	-	x
Phascolarctobacterium	-	-	-	-	-	-	x	-	-	-	-	-
Saccharibacteria_genera	-	-	-	-	-	x	-	-	-	-	-	-
Aquicella	x	-	-	-	-	-	-	-	-	-	-	-
